# A Review of Pityriasis Rosea in Relation to SARS-CoV-2/COVID-19 Infection and Vaccination

**DOI:** 10.7759/cureus.38772

**Published:** 2023-05-09

**Authors:** Nikita Wong, Camilla A Cascardo, Meghan Mansour, Victoria Qian, Geoffrey A Potts

**Affiliations:** 1 Dermatology, Wayne State University School of Medicine, Detroit, USA; 2 Medicine, Oakland University William Beaumont School of Medicine, Rochester, USA

**Keywords:** covid, general dermatology, vaccination, coronavirus, sars-cov-2, covid-19, pityriasis rosea

## Abstract

Pityriasis rosea (PR) is an acute exanthematous disease, commonly preceded by a primary solitary herald patch followed by the onset of smaller scaly papulosquamous lesions within days to weeks. The exact cause of PR remains unclear; however, rash eruptions are thought to be associated with systemic reactivation of human herpesvirus 6 and 7 (HHV-6/7). Several cutaneous manifestations, including PR, have been reported secondary to SARS-CoV-2 infection and/or COVID-19 vaccination. The purpose of this review is to synthesize available data regarding PR in close association with SARS-CoV-2/COVID-19 infection and/or vaccination. A total of 154 patients were included in this study with 62 females and 50 males. PR was reported to occur more commonly in association with SARS-CoV-2/COVID-19 vaccination (102, 66.2%) than during infection (22, 42.3%) or post-infection (30, 57.7%). Interestingly, only 7.1% of patients were tested for concomitant HHV-6/7 past or current infection, with 4.2% testing positive or reporting a history of roseola infantum. While rare, clinicians should be aware of the possibility of patients developing PR associated with SARS-CoV-2/COVID-19 infection and/or vaccination, among other cutaneous reactions. Future studies exploring the link between PR and SARS-CoV-2/COVID-19 infection and/or vaccination would be beneficial, including direct examination of tissue and serological studies for evidence of COVID-19-induced HHV‐6/7 reactivation.

## Introduction and background

Pityriasis rosea (PR) is an acute exanthematous disease, commonly preceded by a primary solitary herald patch followed by the onset of smaller finely scaly erythematous macules or plaques distributed along the trunk and limbs within days to weeks [1]. The exact cause of PR has not been identified; however, epidemiological and clinical features suggest an infective etiology [2]. Several cutaneous manifestations, including PR, have been reported secondary to SARS-CoV-2 infection and/or COVID-19 vaccination [3]. The purpose of this review is to synthesize available data regarding reports of PR eruptions in close association with SARS-CoV-2/COVID-19 infection and/or vaccination.

## Review

**Figure 1:**
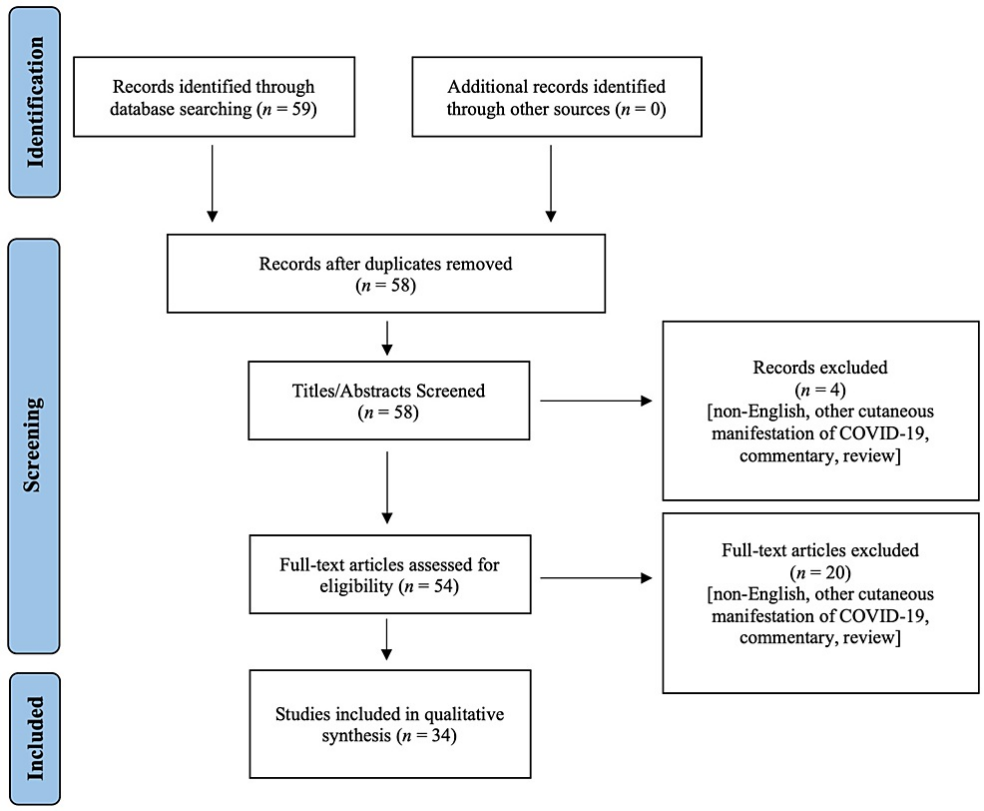
Flow Diagram Based on PRISMA 2020 PRISMA: Preferred Reporting Items for Systematic Reviews and Meta-Analyses, COVID-19: coronavirus disease 2019

We searched PubMed for studies reporting PR cases in relation to SARS-CoV-2/COVID-19 infection and/or vaccination on February 5, 2022. Of the 59 screened studies, 34 met the inclusion criteria, yielding a total of 154 patients (Figure 1). The quality of evidence of the included studies was established using criteria from the 2009 Oxford Levels of Evidence, presented in Table 1.

**Table 1:** Quality of Evidence Established by the 2009 Oxford Levels of Evidence Criteria

Author	Level of Evidence
Abdullah et al. [4]	4
Adya et al. [5]	4
Akdaş et al. [6]	4
Birlutiu et al. [7]	4
Bostan et al. [8]	4
Busto-Leis et al. [9]	4
Carballido Vázquez et al. [10]	4
Català et al. [11]	4
Cohen et al. [12]	4
Cyrenne et al. [13]	4
Dormann et al. [14]	4
Drago et al. [15]	4
Ehsani et al. [16]	4
Gökçek et al. [17]	4
Huang et al. [18]	4
Johansen et al. [19]	4
Leerunyakul et al. [20]	4
Magro et al. [21]	4
Marcantonio-Santa Cruz et al. [1]	4
Martín Enguix et al. [22]	4
Martora et al. [23]	4
Mehta et al. [24]	4
Merhy et al. [25]	4
Mohta et al. [26]	4
Öncü et al. [27]	4
Paolino et al. [28]	4
Pedrazini et al. [29]	4
Piccolo et al. [30]	4
Shin et al. [31]	4
Temiz et al. [32]	4
Veraldi et al. [33]	4
Veraldi and Spigariolo [34]	4
Wang et al. [35]	4
Welsh et al. [36]	4

Sixty-two female (40.3%) and 50 male (32.5%) patients presented with PR in relation to SARS-CoV-2/COVID-19 infection and/or vaccination (Table 2). Of the total number of reports, four included children (<18 years), and the remainder included reports of adults (>/=18 years). PR was reported to occur more commonly in association with the SARS-CoV-2/COVID-19 vaccination (102, 66.2%) than during infection (22, 42.3%) or post-infection (30, 57.7%) (Table 2).

**Table 2:** Characteristics of Pityriasis Rosea Cases Associated With COVID-19 Vaccine Versus Infection *Other: Covishield™, CoronaVac, or Oxford/AstraZeneca **Human herpesvirus 6/7-positive serologies, past history of roseola infantum, or history of roseola infantum contact ***Treatment: topical/oral corticosteroid, oral antihistamine, and/or oral antiviral COVID-19: coronavirus disease 2019

	Number	%
Sex (N=154)		
Female	62	40.3
Male	50	32.5
Not Reported	42	27.3
Age (N=154)		
Children (<18)	4	2.6
Adult (18+)	116	75.3
Not Reported	34	22.1
Herald Patch (N=154)		
Yes	56	36.4
Not Reported	98	63.6
Pityriasis Rosea Eruption With Primary COVID-19 Infection (n=52)		
During COVID-19 Infection	22	42.3
Post-COVID-19 Infection	30	57.7
Pityriasis Rosea Eruption Post-COVID-19 Vaccination (n=102)		
Post-First Dose	46	45.1
Post-Second Dose	38	37.3
Not Reported	18	17.6
Vaccine Manufacturer (n=102)		
Pfizer	42	41.2
Moderna	22	21.6
Johnson & Johnson	0	0.0
Other*	29	28.4
Not Reported	9	8.8
Human Herpesvirus 6 and 7 (N=154)		
Yes**	7	4.5
No	4	2.6
Not Reported	143	92.9
Outcome (N=154)		
Resolved/Improved With Treatment***	75	48.7
Spontaneously Resolved	4	2.6
Recurrence	3	1.9
Not Reported	75	48.7

In patients that reported PR after receiving the SARS-CoV-2/COVID-19 vaccine, Pfizer was the most frequently reported brand received (42, 41.2%), followed by Moderna (22, 21.6%) (Table 2). Patients reported eruption of PR after the first dose of the vaccine and the second dose of the vaccine (46 (45.1%) and 38 (37.3%) cases, respectively) (Table 2). No reports of PR after the booster vaccine or third dose were reported, likely due to the more recent availability of these vaccines. PR appeared an average of 10.2 days (range: 0-30 days) after the administration of the vaccine. Of the reported cases, 56 patients documented the appearance of a herald patch. Only 11 cases had documented serologies or a past medical history of known human herpesvirus (HHV) infection, with seven (4.5%) cases exhibiting positive HHV-6/7 antibodies, a history of roseola infantum, or contact with a person who had roseola infantum and four (2.6%) cases reporting negative serologies (Table 2). Resolution/improvement in PR manifestations after treatment was reported in 75 (48.7%) cases, while four (2.6%) spontaneously resolved; recurrence occurred in three (1.9%) cases (Table 2).

PR is theorized to be a result of the reactivation of HHV‐6 or HHV‐7 [13,37]. Manifestations of PR classically arise with an initial solitary plaque called the “herald patch,” often on the patient’s trunk. In the following days to weeks, the rash generalizes, commonly throughout the trunk and upper arms. This is termed the second eruption, which consists of multiple, discrete, scaly oval plaques and patches along skin cleavage lines [38]. Our data regarding PR in close association with SARS-CoV-2/COVID-19 infection and/or vaccination revealed the appearance of a herald patch in only 36.4% of cases. Alternatively, PR-like eruptions (PR-LE) are typically associated with medications or vaccines. PR-LE do not typically present with a herald patch and instead tend to appear with confluent lesions, intense pruritus, and eosinophilia on histology [13,15]. This atypical presentation is more consistent with the PR described by the literature in association with SARS-CoV-2/COVID-19 infection and/or vaccination; several reports included in this study demonstrated PR with the absence of a herald patch, presence of pruritus, involvement of atypical sites, papulovesicular rash, and associated chilblain‐like lesions. Although the presentation of COVID-19-related PR may be considered atypical, the majority of cases included in this study exhibited susceptibility to conventional supportive therapy with reported resolution of symptoms.

The cases described in this literature review had features of both PR and PR-LE, with neither form presenting more commonly in association with COVID-19 infection or vaccination. Thus, patients who develop a rash after SARS-CoV-2/COVID-19 infection and/or vaccination may present with either PR or PR-LE. Nearly all of the patients included in this review reported resolution of symptoms with supportive therapy, suggesting that either rash eruption should not hinder the continuation of the vaccination series. While the etiology of PR in relation to SARS-CoV-2/COVID-19 infection and/or vaccination remains unknown, of the cases included in this literature review that reported positive or negative serologies, 63.4% of cases described positive HHV-6/7 serologies, a past history of roseola infantum, or contact with a person who had roseola infantum. This supports the theory that SARS-CoV-2/COVID-19 infection and/or vaccination may lead to the reactivation of HHV-6/7 viruses. Català et al. [11] propose that the mechanisms of this reactivation may be due to a strong specific immune response against SARS-CoV-2/COVID-19 infection or the S protein from vaccines diverting cell‐mediated control of another latent virus.

Limitations of this study include the disparateness of the reported data among different manuscripts, as well as the potential exclusion of relevant articles secondary to the search strategy.

## Conclusions

While rare, clinicians should be aware of the possibility of patients developing PR or PR-LE associated with SARS-CoV-2/COVID-19 infection and/or vaccination, among other cutaneous reactions. The management of PR and PR-LE can likely remain supportive as most patients included in this study reported complete resolution of symptoms; however, clinical judgment and patient comfort should ultimately guide this decision. We would like to emphasize that we believe that the protective benefits provided by the SARS-CoV-2/COVID-19 vaccination against infection, hospitalization, and possible death far outweigh the risks of acquiring such cutaneous reactions. Future studies exploring the link between PR and SARS-CoV-2/COVID-19 infection and/or vaccination would be beneficial, including direct examination of tissue and serological studies for evidence of COVID-19-induced HHV‐6/7 reactivation.
